# Characterization of immune cell infiltrate in tumor stroma and epithelial compartments in oral squamous cell carcinomas of Sudanese patients

**DOI:** 10.1002/cre2.501

**Published:** 2021-10-09

**Authors:** Nuha Mohamed Gaafar, Tarig Al‐Hadi Osman, Israa Abdulrahman Ahmed, Mariam Elsheikh, Harsh Dongre, Martha Rolland Jacobsen, Nazar Gafar Mohamed, Siren Fromreide, Ahmed Mohamed Suleiman, Anne Christine Johannessen, Elisabeth Sivy Nginamau, Daniela Elena Costea

**Affiliations:** ^1^ The Gade Laboratory for Pathology, and Centre for Cancer Biomarkers (CCBIO), Department of Clinical Medicine, Faculty of Medicine University of Bergen Bergen Norway; ^2^ Centre for International Health, Department of Global Public Health and Primary Care, Faculty of Medicine University of Bergen Bergen Norway; ^3^ Institute of Clinical Dentistry, Faculty of Medicine University of Bergen Bergen Norway; ^4^ Department of Operative Dentistry University of Science & Technology Omdurman Sudan; ^5^ Department of Oral and Maxillofacial Surgery, Faculty of Dentistry University of Khartoum Khartoum Sudan; ^6^ Khartoum Dental Teaching Hospital Khartoum Sudan; ^7^ Department of Pathology Haukeland University Hospital Bergen Norway

**Keywords:** immunohistochemistry (IHC), oral squamous cell carcinoma (OSCC), toombak, tumor immune microenvironment (TIME)

## Abstract

**Background:**

Tumor immune infiltrate has been explored in oral squamous cell carcinoma (OSCC), but studies on simultaneous characterization of multiple immune cell subtypes separately in stromal and intraepithelial tumor compartments are limited.

**Objectives:**

We aimed to investigate the immune cell infiltrate in OSCC by using immunohistochemistry (IHC) for a panel of inflammatory cells in stromal and epithelial tumor compartments for a better characterization of the tumors.

**Methods:**

Thirty‐six OSCC lesions and nine normal oral mucosa (NOM) samples from patients attending Khartoum Dental Teaching Hospital, Sudan were investigated for presence of tumor infiltrating lymphocytes, tumor‐associated macrophages, tumor‐associated neutrophils, and PD‐L1 positive cells in the inflammatory infiltrate by single and double IHC. Digital quantitative analysis (Aperio Technologies Inc.) was performed separately for stromal and epithelial compartments.

**Results:**

OSCC cases displayed a higher inflammatory infiltrate in the associated stroma, but not in the epithelial compartment when compared to NOM. The immunosuppressive type of inflammatory infiltrate, that is, T regulatory cells (FoxP3^+^ cells) was identified to be significantly higher in the epithelial compartment of tumors with advanced clinical state. An immunoscore developed by combining intraepithelial FoxP3^+^ and CD4^+^ cells was found significantly higher in lesions from elderly patients, localized at toombak dipping‐related sites, poorly differentiated OSCCs, or with loco‐regional lymph node spreading.

**Conclusions:**

Despite heavy immune cell infiltration in tumor‐associated stroma, the majority of OSCCs in this cohort displayed a low intraepithelial immune infiltration. An immunoscore based on combined CD4 and FoxP3 intraepithelial expression may serve as an indicator of advanced tumor progression and should be further investigated for its use as potential prognostic biomarker in OSCC.

## 1 INTRODUCTION

Oral squamous cell carcinoma (OSCC) accounts for more than 90% of oral malignancies, with increasing incidence in developing countries (International Agency for Research on Cancer, 2006). The incidence, prevalence, clinical pattern, and mortality caused by OSCC show geographic variability. In Sudan, OSCC poses a high burden of disease, being characterized by its aggressive behavior and a trend towards increasing incidence, morbidity, and mortality rates (Saeed et al., 2014). Both genetic and environmental factors contribute to OSCC oncogenesis. Several risk factors have been identified such as tobacco use and excessive alcohol consumption (Jiang et al., 2019; Sun et al., 2016). In Sudan, a specific smokeless tobacco called “toombak” is reported to be the main etiologic factor (Patil et al., 2019).

OSCC prognosis remains unsatisfactory. Tumor stratification based on tumor size, regional lymph nodes involvement, and distant metastasis (TNM) along with the histological grading is currently used in the clinical practice. However, it is not sufficient to predict individual prognosis of OSCC (Sano & Myers, 2007). Although different treatment modalities have advanced over the past decade, the 5‐year overall survival (OS) rate of OSCC patients remains low, ~40%–50% (Khan et al., 2019). Locoregional recurrence, metastasis, and second primary tumors are the main factors contributing to the poor OS of OSCC patients (Sekikawa et al., 2019). Thus, there is a pivotal need to identify additional prognostic factors using molecular and clinical markers that would enable more efficient stratifications, which may lead to better prediction of clinical outcome and the appropriate choice of adjuvant therapies. Hanahan and Weinberg pinpointed tumor‐promoting inflammation as one of the enabling hallmarks of cancer essential to carcinogenesis (Hanahan & Weinberg, 2011). Therefore, an improved understanding of the tumor immune microenvironment (TIME), including number, location, and function of infiltrating inflammatory cells is crucial in order to predict disease outcome and to possibly predict the effect of immunotherapeutic strategies, which may prolong the survival of OSCC patients. Several studies investigating simultaneously the presence of different immune cell subtypes indicate that tumor infiltrating lymphocytes (TILs), tumor‐associated neutrophils (TANs), and tumor‐associated macrophages (TAMs) are promising prognostic markers for head and neck cancer patients including OSCC (Lechner et al., 2017; Ryu et al., 2019; Schneider et al., 2018; Stasikowska‐Kanicka et al., 2018). However, these studies have analyzed the lesions as a whole, and only one previous study (Tabachnyk et al., 2012) have explored the TIME separately in tumor‐associated stroma and intraepithelial compartments of OSCCs.

In this study, we aimed at a comprehensive characterization of the inflammatory infiltrate separately in stromal and epithelial tumor compartments, in order to identify putative correlations with clinicopathological features in a prospective cohort of OSCC patients from Sudan selected following REMARK criteria (Sauerbrei et al., 2018). Markers identifying T helper cells (CD4), cytotoxic T cells (CTLs) (CD8), T regulatory cells (T regs) (FoxP3), B‐cells (CD20), macrophages (CD80, CD163, and CD68), and neutrophils (CD66b) were included to investigate these cell subsets in tumor‐associated stroma and their intra‐tumoral infiltration, in addition to PD‐L1 biomarker chosen as a surrogate marker for activation of the tumor suppressive mechanism. We hypothesize that density and spatial distribution of the selected immune cell subsets are different in the stromal and epithelial compartments of tumors, and it can be used as an indicator of tumor progression.

## 2 MATERIALS AND METHODS

### 2.1 Patient cohort

Ethical approval for this study was obtained from National Health Research Ethics Committee in Sudan (*FMOH/RD/SEC/09*) and Norwegian Regional Committee for Medical and Health Research Ethics (*REKVest 3*.*2006*.*2620*, *REKVest 3*.*2006*.*1341*). Thirty‐six patients diagnosed with primary OSCC between November 2014 and June 2015 at Khartoum Dental Teaching Hospital (KDTH), Sudan, the main referral center for head and neck cancer in Sudan, were included prospectively together with nine non‐cancer patients with no oral mucosal conditions that were attending KDTH for removal of wisdom teeth (total *N* = 45). All patients and controls had signed an informed consent before participating to the study.

### 2.2 Immunohistochemistry

Sections of 3–4 μm thickness were cut and prepared from each formalin‐fixed paraffin‐embedded (FFPE) tissue block using microtome (Leica Microsystems, Germany) and were mounted on coated glass slides (Thermo Scientific Superfrost Plus, USA). Slides were incubated at 56°C for 24 h and then stored at 4°C for maximum of 1‐month duration before further immunostaining. The single and double staining methods were performed as previously described and are presented in more detail in Supporting Information (Osman et al., 2013). The antibodies used in this study are presented in Table S1 to identify the main subsets of inflammatory cells such as: T helper cells, CTLs, T regs, B‐cells, TAMs, and TANs in addition to PD‐L1 biomarker. TILs and TAMs were chosen to investigate the chronic inflammation while TANs were chosen to investigate active inflammation.

### 2.3 Slide scanning and annotation

Digital scanning of stained tissues was carried out at 40× magnification using a whole slide scanner (Hamamatsu NanoZoomer‐XR, Japan). Image Scope software (Aperio Technologies Inc., Vista, CA) was used to visualize the scanned slides. Areas of analyses were manually annotated on the scanned sections by choosing five to seven hot spots (i.e., the densest and more intensely stained areas with a collection of minimum 15 stained cells; Jang et al., 2017).

**Figure 1:**
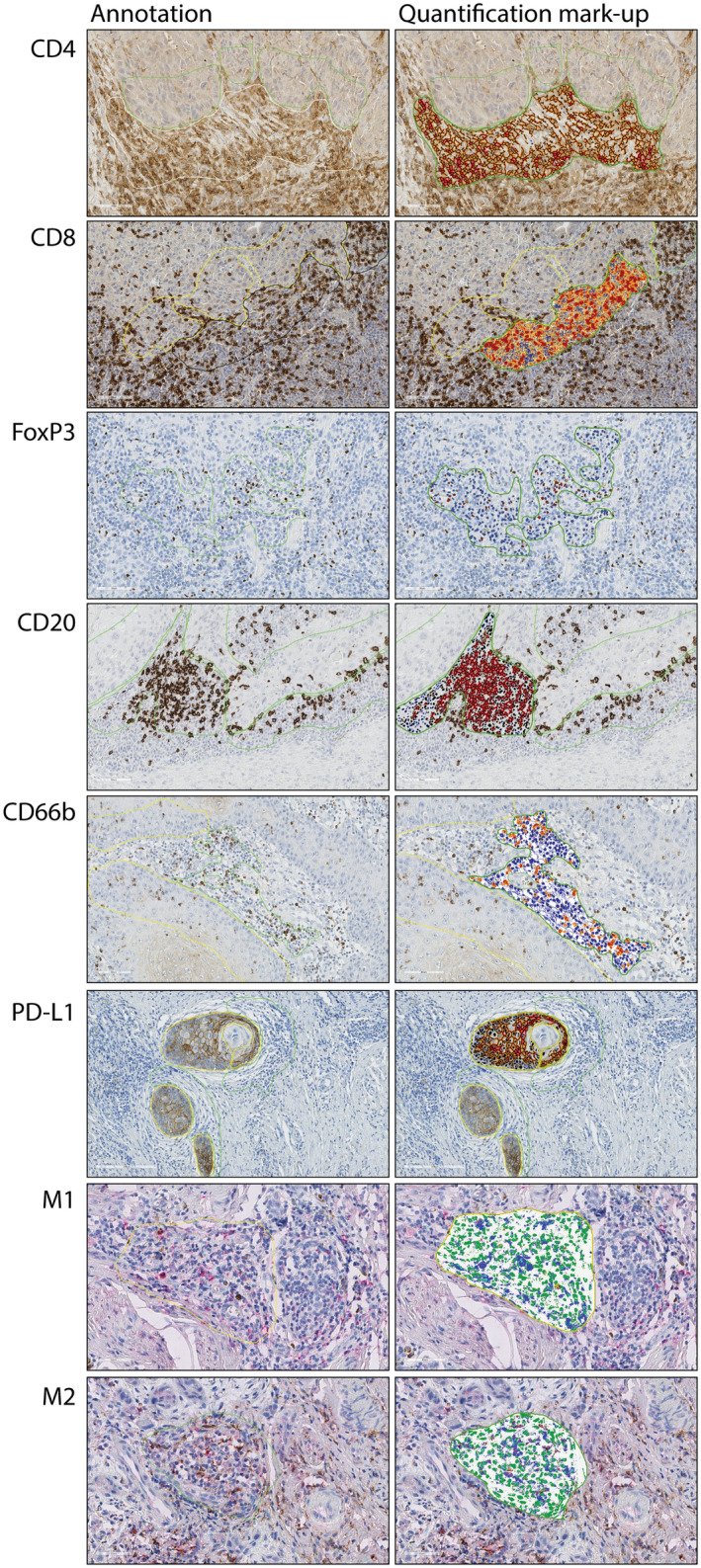
Representative images of IHC staining in OSCC, showing hot spots and the specific annotation algorithm mark‐up for each biomarker

Annotations were drawn on central areas of tumors, separately on stromal and epithelial compartments. Exploration of pan CK‐stained serial slides was done prior to annotations for each case, as a preparation for accurate identification of the two tumor compartments. For NOM sections, annotations were drawn within normal squamous epithelium and subepithelial connective tissue. Overall, an annotation pair was obtained for each marker in OSCC and NOM groups separately, and for each of those in epithelial and stromal compartment. The following criteria were pursued to standardize the annotating procedure: annotations' width was set to a maximum of 100 μm from the epithelial–mesenchymal interface towards both epithelial and stromal compartments (Figure 1); when found, hot spot areas were preferentially selected with a minimum of 0.2 mm^2^, drawn as total annotated area; single scattered fat cells were included but areas with fat, muscles, vessels, and nerves were excluded.

### 2.4 IHC staining quantification

To analyze the selected annotations, several Aperio algorithms were selected and optimized (Aperio Technologies Inc., Vista, CA). They were adjusted based on red, green, and blue (RGB) values, detection threshold, exclusion of background staining, and cell size and shape to achieve a representative mark‐up image of the staining. Aperio Nuclear v9 algorithm was chosen to detect anti‐FoxP3 chromogenic staining and count FoxP3^+^ stained cells. The cells were counted as positive with staining intensities 1+, 2+, and 3+, while cells with staining intensity 0 were considered negative. For CD4, CD20, and PD‐L1, Aperio membrane v9 algorithm was selected to detect the chromogenic staining and count cells with membranous staining. Cells with 0 value were considered negative, while the rest were categorized as positive. Aperio color deconvolution v9 algorithm was selected to detect CD8 and CD66b chromogenic staining and to calculate CD8^+^ and CD66b^+^ pixels in the specified analyzed area. Aperio colocalization v9 Algorithm was chosen to detect M1 (CD80/CD68) and M2 (CD163/CD68) staining and calculate M1^+^ and M2^+^ pixels of DAB and LPR staining in the specified analyzed area.

### 2.5 Data presentation and statistical analysis

For FoxP3, CD4, CD20, and PD‐L1, the output was expressed as number of positive cells/total analyzed area. For CD8 and CD66b, data output were expressed as (total stained area/total analyzed area) ×100. Lastly, M1 and M2 data output were expressed as (percentage of positive DAB pixels/percentage of LPR positive pixels)/total analyzed area.

All statistical analysis was performed using SPSS 25.0 software (SPSS, Chicago, IL). Before starting any statistical analysis, histogram, test of normality, skewness, and Q–Q plots were applied to test the distribution of all data. Accordingly, either parametric or nonparametric tests were applied. Protein expression difference between stromal and epithelial compartments of OSCC was investigated using paired *t*‐test for CD4, CD8, and CD20, while related Mann–Whitney *U* test was used for FoxP3, CD66b, PD‐L1, M1, and M2. OSCC and NOM difference in protein expression was analyzed by independent sample *t*‐test for CD4 and CD8 for both stromal and epithelial compartments, while the difference was assessed by using Mann–Whitney *U* test for FoxP3, CD20, CD66b, PD‐L1, M1, and M2 in both tumor‐associated stroma and intraepithelial infiltration.

Data were stratified according to demographic and clinicopathological variables. The median values obtained for the whole cohort and for each marker (each type of immune cell) were used as the cut‐off value to dichotomize patients' tissues into groups with high and low infiltration. Chi square test (*χ* ^2^) was used to investigate correlations between biomarkers and between each marker expression in stroma and epithelium. All tests were two‐sided and *p* < 0.05 was considered statistically significant.

## 3 RESULTS

### 3.1 Cohort demographics

A total number of 45 participants, 36 OSCC patients, and 9 NOM of non‐cancer controls, were included in this study. Mean age of control group was 46.11 ± 10.01 years (median = 44.00 years) with a range from 39 to 70 years. The age for OSCC group was more spread (range 25–83 years) and the mean was higher (mean = 62.31 ± 11.85 years, median = 65.00 years) than of controls. Males represented 3/9 (33.3%) of control group and 24/36 (66.7%) of OSCC group. Numbers of missed and decayed teeth were higher in OSCC group than in non‐cancer controls. Number of individuals with poor oral hygiene was higher in OSCC (41.7%) than non‐cancer controls (33.3%; Table 1).

**Table 1:** Demographic and clinical characteristics of the study groups

Clinical features	OSCC (*n* = 36) *N* (%)	NOM (*n* = 9) *N* (%)
Age		
≥65 years	19 (47.2)	1 (11.1)
<65 years	17 (52.8)	8 (88.9)
Gender		
Male	24 (66.7)	3 (33.3)
Female	12 (33.3)	6 (66.7)
Diabetes		
Yes	3 (8.3)	0 (0.0)
No	33 (91.7)	9 (100.0)
Systemic disease		
Yes	5 (13.9)	1 (11.1)
No	31 (86.1)	8 (88.9)
Smoking		
Yes (past and/or current smoker)	13 (36.1)	1 (11.1)
No	23 (63.9)	7 (77.8)
Toombak		
Yes (past and/or current user)	16 (44.4)	0 (0.0)
No	20 (55.6)	8 (88.9)
Alcohol		
Yes (past and/or current user)	7 (19.4)	0 (0.0)
No	27 (75.0)	8 (88.9)
Missed teeth (MT) according to population median		
Low	16 (44.4)	6 (66.7)
High	18 (50.0)	3 (33.3)
Decayed teeth (DT) according to population median		
Low	19 (52.8)	6 (66.7)
High	15 (41.7)	3 (33.3)
Community periodontal index for treatment needs (CPITN)		
1‐CPI	5 (13.9)	5 (55.6)
2‐CPI	11 (30.6)	3 (33.3)
3‐CPI	14 (38.9)	0 (0.0)
Gingival index (GI)		
1‐GI	8 (22.2)	6 (66.7)
2‐GI	21 (58.3)	1 (11.1)
3‐GI	2 (5.6)	1 (11.1)
Oral health index simplified (OHIS)		
1‐OHIS	1 (2.8)	0 (0.0)
2‐OHIS	15 (41.7)	5 (55.6)
3‐OHIS	15 (41.7)	3 (33.3)

Abbreviations: NOM, normal oral mucosa; OSCC, oral squamous cell carcinoma.

In OSCC group, a history of smoking (36.1%), toombak use (44.4%), and alcohol consumption (19.4%) was self‐reported by the patients. Lesions were located mainly at typical snuff dipping sites, that is, sulcular, labial, and buccal mucosa (52.8%). Majority of patients were diagnosed with an advanced clinical stage (TNM clinical stage III/IV = 88.9%) and accordingly, most of lesions were large‐sized tumors (61.2%). Lymph node involvement was reported in 77.8%. One‐third (33.4%) of tumors were well‐differentiated (Table 2).

**Table 2:** Clinical and histological characteristics of the OSCC cases

Clinical and microscopic features	OSCC *N* (%)
Location	
Buccal, labial, and sulcular	19 (52.8)
Alveolar, palate, and retromolar	11 (30.6)
Tongue	3 (8.3)
Tumor size	
T1	2 (5.6)
T2	8 (22.2)
T3	11 (30.6)
T4	11 (30.6)
Lymph nodes involvement	
N0	3 (8.3)
N1	14 (38.9)
N2	13 (36.1)
N3	1 (2.8)
Tumor stage	
I	0 (0.0)
II	2 (5.6)
III	11 (30.6)
IV	21 (58.3)
Tumor grading	
Well‐differentiated	12 (33.3)
Moderately differentiated	16 (44.4)
Poorly differentiated	8 (22.2)

### 3.2 Higher inflammatory cell infiltrate in stromal compartment than in epithelial tumor compartment

All inflammatory cell types were present in both normal controls and OSCC cases, both in stromal and epithelial compartment, with few exceptions. In the connective tissue of NOM, 88.9% of cases displayed FoxP3^+^ cells and only 11.1% of cases showed PD‐L1^+^ staining, M1 and M2 macrophage phenotypes. Normal epithelium of non‐cancer patients displayed also FoxP3^+^ cells in 88.9% of the specimens, PD‐L1^+^ cells in 44.4% of specimens, and no M1 and M2 macrophage phenotypes in any of the specimens.

In contrast, only 97.2% of OSCC cases showed presence of FoxP3^+^ and M1 phenotype staining in stromal compartment while 86.1% showed staining for PD‐L1. For epithelial compartment, 97.2% of OSCC cases showed presence of FoxP3^+^ and CD20^+^ cells while 72.2% showed presence of PD‐L1^+^ cells (Table S2).

**Figure 2:**
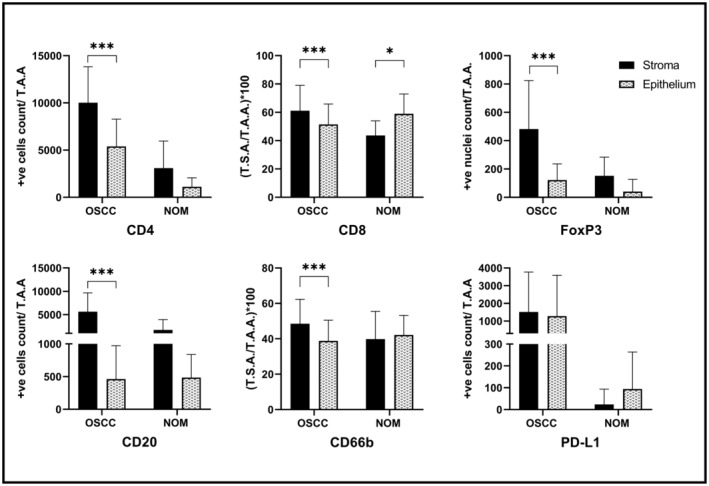
Quantification of biomarkers' expression in stromal and epithelial compartments of OSCC and NOM

For OSCC cases, the inflammatory infiltrate was generally more abundant in stromal than epithelial compartment. This difference was statistically significant for all biomarkers, except PD‐L1 that was present at similar levels and distribution in both compartments (Figure 2). The overall picture was more complex and variable in normal controls. T helper, T reg, and B‐cell subsets showed the same trend, with higher infiltration in connective tissue compartment than intraepithelially, but an opposite trend was observed for CTLs, TANs and PD‐L1, with higher expression intraepithelially than in stromal compartment, although not statistically significant except only for CTLs (Table S3).

### 3.3 Higher infiltration of immune‐suppressive cell phenotypes in epithelial tumor compartment than in normal oral epithelium

**Figure 3:**
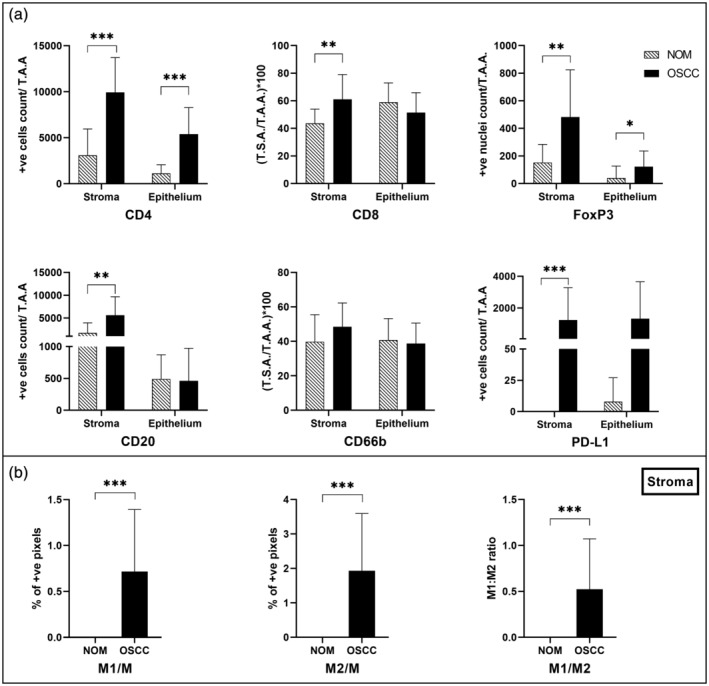
(a) Quantification of biomarkers' expression in NOM and OSCC. In both compartments; (b) quantification of macrophages in the stromal compartment only

Quantitative analysis showed that OSCCs was significantly higher infiltrated by inflammatory cells (i.e., TILs and TAMs) in tumor‐associated stroma compared to connective tissue of NOM for all biomarkers except CD66b, where the difference was not statistically significant (Figure 3; Table S4).

For epithelial compartment, a more heterogeneous picture was observed. The same trend of higher expression in OSCC than NOM was observed for CD4^+^ (*p* < 0.001), FoxP3^+^ (*p* = 0.012), and PDL1^+^ cells (*p* = 0.100). The opposite trend, with more infiltrates in NOM than in the OSCC group was observed for CD8, CD20, and CD66b markers, although not statistically significant (Figure 3; Table S4).

### 3.4 Correlations between clinicopathological features and density of immune cells

**Figure 4:**
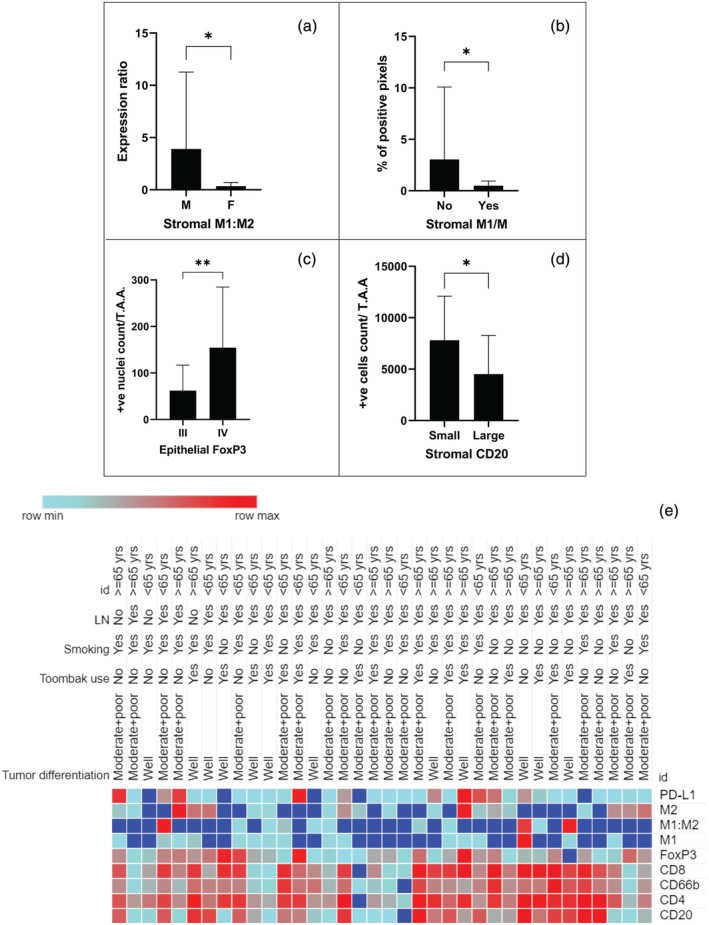
Graphs of biomarkers significantly correlated with (a) gender, (b) alcohol consumption, (c) tumor stage, (d) tumor size; (e) a heat map showing OSCC stratification in the stromal compartment according to clinicopathological features of the cohort. Age is labeled as id

Higher intraepithelial tumor infiltration with T regs was associated with advanced clinical state of tumors (*p* = 0.009, Figure 4) and with moderately and poorly differentiated tumors (*p* = 0.001). B‐cell infiltration in tumor‐associated stroma was higher in small‐size tumors (*p* = 0.035) than in large tumors, as shown in Figure 4. No association was found between lymph node involvement and inflammatory cell infiltration.

A tendency for a higher lymphocytic and granulocytic infiltration according to tobacco use, either smoked or smokeless, was also observed, although not statistically significant (Figure 4e).

### 3.5 Correlations between different immune cell infiltrates in stromal and epithelial compartments

A strong positive correlation was observed between presence of T helper cells, CTLs, B‐cells, and TANs in stromal compartment of OSCC. An inverse moderate correlation was found between T helper cells and M2 phenotype. TANs showed a moderately positive correlation with M1:M2 ratio, while there was an inverse moderate correlation with M2 phenotype (Table S5).

In epithelial compartment, presence of B‐cells displayed a moderate correlation with presence of CTLs, T regs, and PD‐L1^+^ cells (Table S6).

The amount of infiltrates in stromal and epithelial compartment showed strong correlation for T regs (phi = 0.83, *p* < 0.001), and moderate correlation for CTLs (phi = 0.53, *p* = 0.001), B‐cells (phi = 0.62, *p* < 0.001), TANs (phi = 0.46, *p* = 0.006), and PD‐L1^+^ cells (phi = 0.33, *p* = 0.048).

### 3.6 Immunoscore

An immunoscore was calculated by multiplying statistically significantly different inflammatory biomarkers observed in epithelial compartment of OSCC when compared to NOM, namely CD4 and FoxP3. The resulted immunoscore showed statistically significant higher intraepithelial infiltration in elderly patients' group when compared to younger patients' group (*p* = 0.031), and in tumors of patients with lymph nodes involvement than those with no lymph node metastasis (*p* = 0.006). This score also showed higher inflammatory cell intraepithelial infiltration in moderately and poorly differentiated OSCCs than in well‐differentiated OSCCs (*p* = 0.005). Higher intraepithelial infiltration was found in OSCCs localized at toombak‐dipping sites (i.e., sulcular, labial, and buccal mucosa) than those OSCCs located at other sites (*p* = 0.019).

## 4 DISCUSSION

One of the major findings of this study is that the inflammatory cell infiltration was more abundant in tumor‐associated stroma than intraepithelial compartment, resulting in low stroma to epithelial compartment ratio of infiltration. This is the picture of immune‐excluded tumors, that is, with immune cells aggregating at the tumor boundaries and low density of infiltrate inside the tumor (Kather et al., 2018; Li et al., 2019; Thorsson et al., 2018). Since this study was performed on a cohort with approximately half of patients presenting with the habit of toombak dipping with OSCC lesions localized mainly in the dipping sites of the buccal vestibulum, it might indicate that toombak‐related OSCCs belong to the group of immune‐excluded tumors. In this cohort, the use of toombak was assessed based on self‐reporting, but further supporting this theory is also the fact that a higher immunoscore build on the combination of T helper and T reg scores was associated to localizations of OSCC lesions related to toombak‐dipping sites.

It is notable that although OSCC showed denser immune cell infiltration in tumor‐associated stroma than in connective tissue of NOM, there was less infiltration of anti‐tumor CTLs, B‐cells, and TANs in the intraepithelial compartment of OSCC than in the epithelium of NOM. The higher immune cells recruitment in stroma, in line with previous studies (Stasikowska‐Kanicka et al., 2018; Tabachnyk et al., 2012) was thus not reflected in higher intraepithelial infiltrates. One might speculate that this highlights the role of stromal microenvironment in attracting and modulating inflammatory cells in OSCC, as previously suggested by others (Takahashi et al., 2017). However, higher T helper cell infiltrates are in our cohort inversely correlated to M2 type CD68 positive cells, and a trend is seen towards a positive correlation with T reg positive cells, which is in line with one previous study (Moreira et al., 2010). It would be interesting in the future to investigate which pathways regulate recruitment and/or expansion of T regs to tumoral epithelia. Furthermore, while FoxP3 could predict a more advanced disease in our cohort, it is important to keep in mind that most of the included patients had clinical stage III/IV disease at the time of surgery, and hence our results might not represent the cell interactions in TIME at early stages.

In agreement with this observation, we found an increased B‐cell stromal infiltration in small tumors compared to large tumors. Since tumor size affects staging of tumors and prognosis, our finding may indicate a potential positive role of B‐cells in TIME in early stages. Although the sample size of our cohort is quite small, it shows trends that are in line with previous studies on larger cohorts. B‐cell presence has been associated with positive clinical outcome in head and neck cancer (Pretscher et al., 2009; van Herpen et al., 2008), and increased infiltration of CD20^+^ B‐cells in TIME of OSCC was significantly correlated with improved survival (Wirsing et al., 2018).

Immune escape mechanisms allow cancer cells to escape from the attack of the immune system and are thought to be determinants of tumor progression in both T cell–inflamed and non‐inflamed tumors. In this study, we found that a denser PD‐L1^+^ cell inflammation was present in the tumor stroma when compared to the connective tissue stroma under normal mucosa indicating the activation of immune escape mechanisms in OSCC tumors. The amount of PD‐L1+ inflammatory cells varied widely between cases. However, we could not detect any significant correlations with other clinical and histopathological parameters.

TILs and TANs were found in all tumors investigated here in aggregates, which is in line with most recent published studies (Caldeira et al., 2017; Fang et al., 2017; Taghavi et al., 2018). A trend was observed of increased TANs infiltration in tumors of patients with severe gingivitis. Although this was not statistically significant, it may indicate an association between tumor innate immunity and gingival inflammatory status. Further investigation is needed to confirm this observation. In addition, we also identified a strong correlation within all TILs subsets but T reg positive ones and TANs, indicating possible functional agreement among those subsets, and suggesting a possible effect of the oral microbiome on the tumor response.

The presence of a higher inflammatory infiltrate in OSCC has been suggested for more than three decades ago as an indicator of good prognosis (Bryne et al., 1989; Bryne et al., 1992). Here, we went into a more in‐depth analysis of the inflammatory infiltrate. A known method developed to quantify the in situ immune infiltrate is the “immunoscore.” (Galon et al., 2014; Galon & Bruni, 2019) It was first developed as a scoring system based on the quantification of two TIL subsets (CD3 and CD8). Here, we developed a score based on quantification of two TIL subsets: T helper and T reg (CD4 and FoxP3) lymphocyte subpopulations, significantly different in intraepithelial tumor component when compared to NOM. We found higher score in elderly patients (65+ years), in tumors with lymph node involvement, in poorly differentiated and in toombak‐related OSCCs. These associations suggest that CD4 and FoxP3 biomarkers may be used as a measure of a pro‐tumor immune infiltrate.

This study may help to generate other hypotheses which can be tested on bigger cohorts as the sample size is the main limitation of the current study. Another limitation is the divergence between controls and OSCC cases in terms of age and gender. However, the NOM participants were selected from the same hospital and share the same habits and socioeconomic status with OSCC patients.

## 5 CONCLUSIONS

Despite overall increase of immune cell infiltration in the tumor stroma, majority of cases of this toombak‐related cohort of OSCCs displayed lower numbers of anti‐tumor and higher infiltration of immune‐suppressive cell phenotypes in epithelial tumor compartment than in normal oral epithelium. An immunoscore based on combined CD4 and FoxP3 intraepithelial expression may serve as an indicator of tumor progression and should be further investigated for its use as a potential prognostic biomarker in OSCC. Distinctive composition of immune infiltrate in stromal and epithelial compartments points also towards possible different regulatory mechanisms of immune response against tumor between the two compartments and at different stages of tumor growth.

## CONFLICT OF INTEREST

The authors declare no conflict of interest.

## AUTHOR CONTRIBUTIONS

Nuha Mohamed Gaafar, Tarig Al‐Hadi Osman, Anne Christine Johannessen, Elisabeth Sivy Nginamau, and Daniela Elena Costea have conceived the study. Nuha Mohamed Gaafar, Israa Abdulrahman Ahmed, Mariam Elsheikh, Harsh Dongre, Martha Rolland Jacobsen, Nazar Gafar Mohamed, and Siren Fromreide performed experiments. Israa Abdulrahman Ahmed, Nuha Mohamed Gaafar, and Mariam Elsheikh collected the patient material. Nuha Mohamed Gaafar and Tarig Al‐Hadi Osman analyzed data. Nuha Mohamed Gaafar prepared the figures and the manuscript. Tarig Al‐Hadi Osman, Elisabeth Sivy Nginamau, Anne Christine Johannessen, and Daniela Elena Costea were supervising the work. All authors have participated to the writing and editing of the manuscript and approved the submitted version.

## Supporting information


**Appendix** **S1**. Supplementary Materials and Methods

## Data Availability

Data available on request from the authors.
