# Supplementary material for: Characterization of immune cell infiltrate in tumor stroma and epithelial compartments in oral squamous cell carcinomas of Sudanese patients
Source: Clin Exp Dent Res. 2021 Oct 9;8(1):130–40. doi: 10.1002/cre2.501 (PMC8874073; doi:10.1002/cre2.501)
Supplement: Supplementary file 1 — Appendix S1. Supplementary Materials and Methods [file CRE2-8-130-s001.docx]

**Supplementary material**

**A. Supplementary Materials and Methods**

**Patient cohort**

Inclusion criteria were; age ≥ 18 years with no previous history of malignancy, enough tissue for analyses, and for OSCC patients only, no history of adjuvant chemo- or radiotherapy received before surgical resection. All patients who were seropositive for human immunodeficiency virus (HIV) or hepatitis B surface antigen (HBsAg) were excluded. Patients were clinically examined and interviewed by a team that obtained disease history and follow-up information from medical records. An informed written consent was obtained from all patients before enrollment in the study. For dental variables, decayed and missed teeth (DMT) were calculated, recorded and categorized into high and low, according to population median based on a survey of oral health in a Sudanese population^(1)^. Community periodontal index of treatment needs (CPITN) was recoded and categorized according to the British Society of Periodontology criteria^(2)^. Gingival index (GI) and the simplified oral hygiene (OHIS) indices were recorded according to WHO criteria^(3)^.

**Single staining**

All sections were deparaffinized by immersion in xylene for 10 min and rehydrated for 15 min by immersion in graded alcohol concentrations (100%, 96% and 70%). Epitope retrieval was optimized individually for each biomarker (Table S1). Heat-induced epitope retrieval was performed in a microwave oven (Whirlpool, MI, USA) for 25 min in total, first at 950 W for 7-8 min until the retrieval solution boiled and then at 350 W for 17-18 min. Two different in-house retrieval solutions were used; Citrate (2.1 g citric acid (VWR International, PA, USA), 9 ml 3N NaOH (Sigma-Aldrich, MO, USA), 1 L milliQ water, pH 6.0) and Tris/EDTA (1.205 g Tris-base (Sigma-Aldrich, MO, USA), 0.395 g EDTA (VWR International, PA, USA), 1 L milliQ water, pH 9.0). Blockage for the unspecific binding sites was done for 30 min with a solution of 10% normal goat serum (Agilent Technologies, Golstrup, DK) and 3% bovine serum albumin (Agilent, CA, USA) in Tris-buffered saline (TBS) (Sigma-Aldrich, MO, USA).

Primary antibody incubation was performed for detection of CD4, CD8, CD20, CD66b, FoxP3, PD-L1, and PanCK antigens for 1 hr at room temperature (RT) as described in Table S1, with inclusion of negative controls and appropriate positive controls. EnVision™ visualization system (Agilent Technologies) was used for all antibodies except for PanCK, where MACH3 (Biocare Medical, CA, USA) was used. The visualization was performed as follows: endogenous peroxidase in sections was inactivated by incubating with hydrogen peroxide for 5 min. Sections were then incubated for 30 min with horseradish peroxidase (HRP) conjugated anti-mouse or rabbit secondary antibody. Thereafter, sections were incubated with diaminobenzidine (DAB) chromogen for 10 min. Counterstaining was made by incubation in haematoxylin for 5 min. At last, sections were dehydrated with graded alcohol concentrations (70%, 96% and 100%) followed by immersion in xylene for 4 min and covered immediately.

**Double staining**

Sections were incubated overnight with the first primary antibodies (CD80 or CD163) at 4 °C. Then, the reaction was detected by DAB chromogen as described before. Thereafter, an additional denaturation step was performed in order to block antibody cross-reactivity using the heat-induced method for 8 min (at 950 W in pH 6.0 retrieval solution for 3 min and then at 350 W for 5 min)^(4)^, and sections were allowed to cool. Then, sections were incubated with the second primary antibody (CD68) for 1h at RT. EnVision™ Double stain system (Agilent Technologies) was used for the remaining steps as follows: reactions from the primary antibodies were blocked for 3 min by double stain block and for 10 min with dual block to prevent the cross reactivity with the residual tissue alkaline phosphatase (AP). To visualize the second primary antibody, sections were incubated with labelled AP anti-mouse for 45 min. Staining for the second primary antibody was developed by incubation with the liquid permanent Red (LPR) substrate-chromogen for 8 min. Slides were counterstained, covered and mounted as indicated for single staining.

The IHC assay was monitored and controlled for both false positive and negative reactions. Tonsil, placenta, known positive OSCC for selected markers and oral epithelial tissue sections were used as positive assay control based on suppliers’ recommendations (Table S1). Negative assay control was performed by using sections incubated without adding the primary antibody.

**References**

1. Khalifa N, Allen PF, Abu-bakr NH, Abdel-Rahman ME, Abdelghafar KO. A survey of oral health in a Sudanese population. BMC Oral Health. 2012;12:5.

2. Ainamo J, Barmes D, Beagrie G, Cutress T, Martin J, Sardo-Infirri J. Development of the World Health Organization (WHO) community periodontal index of treatment needs (CPITN). Int Dent J. 1982;32(3):281-91.

3. Loe H. The Gingival Index, the Plaque Index and the Retention Index Systems. J Periodontol. 1967;38(6):Suppl:610-6.

4. Osman TA, Oijordsbakken G, Costea DE, Johannessen AC. Successful triple immunoenzymatic method employing primary antibodies from same species and same immunoglobulin subclass. Eur J Histochem. 2013;57(3):e22.

**B. Supplementary Figure**

**
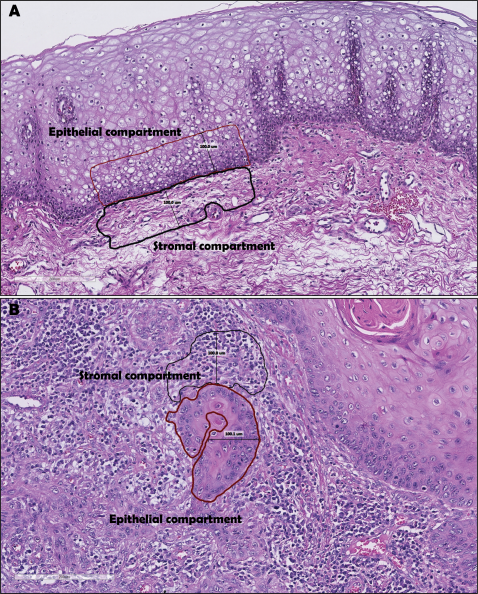
**

**Supplementary Figure S1** Representative image (HE staining) showing the epithelial and stromal compartments in NOM (A) and OSCC (B). Notice the immune cell infiltration in the stroma.

**C. Supplementary Tables**

**Table S1** Antibodies and conditions used for IHC assays

| Primary antibody and specificity | Clone | Dilution | Retrieval solution | Temperature | Incubation | Positive control | Source |
| --- | --- | --- | --- | --- | --- | --- | --- |
| Monoclonal Mouse Anti-human CD4 | 4B12 | 1:20 | Tris/EDTA pH=9 | RT | 1h | Tonsil | Agilent (USA) |
| Monoclonal Mouse Anti-human CD8 | C8/144B | 1:100 | Tris/EDTA pH=9 | RT | 1h | Tonsil | Agilent (USA) |
| Monoclonal Mouse Anti-human FoxP3 | 259D/C7 | 1:500 | Citrate pH=6 | RT | 1h | OSCC | BD Pharmingen™ (Canada) |
| Monoclonal Mouse Anti-human CD20cy | L26 | 1:3000 | Tris/EDTA pH=9 | 4֯ C | ON | Tonsil | Agilent (USA) |
| Polyclonal Rabbit Anti-human CD66b | - | 1:300 | Citrate pH=6 | RT | 1h | OSCC | Abcam (UK) |
| Monoclonal Rabbit Anti-human PD-L1 | EPR19759 | 1:250 | Citrate pH=6 | RT | 1h | Placenta & OSCC | Abcam (UK) |
| Monoclonal Mouse Anti-human CD68 | PG-M1 | 1:150 | Citrate pH=6 | RT | 1h | Tonsil | Agilent (USA) |
| Monoclonal Mouse Anti-human B7-1 (CD80) | 37711 | 1:200 | Citrate pH=6 | 4֯ C | ON | Tonsil | R&D Systems (Germany) |
| Monoclonal Mouse Anti-human CD163 | 10D6 | 1:600 | Citrate pH=6 | 4֯ C | ON | Tonsil | Abcam (UK) |
| Monoclonal Mouse Anti-human PanCytokeratin | MNF116 | 1:1000 | Proteinase K | RT | 1h | Oral epithelial tissue | Agilent (USA) |

RT: room temperature, ON: Overnight

**Table S2** Mean, median and standard deviation of tumor inflammatory biomarkers in the OSCC samples.

| Biomarker | Location | Mean | median | SD | Range |
| --- | --- | --- | --- | --- | --- |
| CD4 | Stroma | 9933.75 | 10554.27 | 3786.42 | 347.20 -15779.37 |
|  | Epithelium | 5388.66 | 4926,20 | 2892.32 | 33.25 – 11181.67 |
| CD8 | Stroma | 61.13 | 65.33 | 17.94 | 26.56 – 87.32 |
|  | Epithelium | 51.50 | 49.82 | 14.40 | 30.45 – 84.46 |
| FoxP3 | Stroma | 481.90 | 444.67 | 342.89 | 0.00 – 1358.08 |
|  | Epithelium | 122.01 | 97.69 | 114.58 | 2.90 – 506.42 |
| CD20 | Stroma | 5632.62 | 5587.91 | 4020.08 | 12.64 – 12613.15 |
|  | Epithelium | 461.68 | 328.15 | 510.15 | 0.00 – 2579.22 |
| CD66b | Stroma | 48.53 | 50.58 | 13.76 | 21.53 – 82.96 |
|  | Epithelium | 38.85 | 36.17 | 11.63 | 23.39 – 74.95 |
| PD-L1 | Stroma | 1516.95 | 187.10 | 2250.65 | 0.00 – 7513.40 |
|  | Epithelium | 1284.34 | 56.66 | 2308.85 | 0.00 – 7953.87 |
| M1/M | Stroma | 1.58 | 0.72 | 3.08 | 0.00 – 18.22 |
| M2/M |  | 2.14 | 1.21 | 2.32 | 0.06 – 9.20 |
| M1:M2 |  | 2.72 | 0.35 | 6.21 | 0.00 – 25.53 |

**Table S3** Comparison of biomarkers’ expression in the stromal compartment and epithelial compartment.

| Marker | Group | Stroma | | Epithelium | | *P* value | Direction |
| --- | --- | --- | --- | --- | --- | --- | --- |
|  |  | **Mean** | **SD** | **Mean** | **SD** |  |  |
| CD4 | OSCC | 10021.78 | 3804.15 | 5388.66 | 2892.32 | < 0.001 | Stroma > epi |
| CD8 | OSCC | 61.13 | 17.94 | 51.50 | 14.40 | < 0.001 | Stroma > epi |
| FoxP3 | OSCC | 481.90 | 342.80 | 122.01 | 114.58 | < 0.001 | Stroma > epi |
| CD20 | OSCC | 5632.62 | 4020.08 | 461.68 | 510.15 | < 0.001 | Stroma > epi |
| CD66b | OSCC | 48.53 | 13.76 | 38.85 | 11.63 | < 0.001 | Stroma > epi |
| PD-L1 | OSCC | 1516.95 | 2250.65 | 1284.34 | 2308.85 | 0.489 | Stroma > epi |
| CD4 | NOM | 3093.28 | 2869.09 | 1123.29 | 947.82 | 0.086 | Stroma > epi |
| CD8 | NOM | 43.62 | 10.36 | 58.94 | 14.03 | 0,017 | Epi > stroma |
| FoxP3 | NOM | 152.22 | 131.60 | 40.76 | 85.97 | 0.116 | Stroma > epi |
| CD20 | NOM | 1726.27 | 2212.58 | 486.32 | 352.14 | 0.093 | Stroma > epi |
| CD66b | NOM | 39.83 | 15.67 | 42.17 | 11.06 | 0.674 | Epi > stroma |
| PD-L1 | NOM | 23.38 | 70.15 | 94.12 | 169.64 | 0.109 | Epi > stroma |

**Table S4** Comparison of biomarkers’ expression between NOM and OSCC groups

| Marker | Location | NOM | | OSCC | | *P* value | Direction |
| --- | --- | --- | --- | --- | --- | --- | --- |
|  |  | **Mean** | **SD** | **Mean** | **SD** |  |  |
| CD4 | Stroma | 3093.28 | 2869.09 | 9933.75 | 3786.42 | < 0.001 | OSCC > NOM |
| CD8 | Stroma | 43.62 | 10.36 | 61.13 | 17.94 | 0.001 | OSCC > NOM |
| FoxP3 | Stroma | 152.22 | 131.60 | 481.90 | 342.89 | 0.004 | OSCC > NOM |
| CD20 | Stroma | 1726.27 | 2212.58 | 5632.62 | 4020.08 | 0.001 | OSCC > NOM |
| CD66b | Stroma | 39.83 | 15.67 | 48.53 | 13.76 | 0.155 | OSCC > NOM |
| PD-L1 | Stroma | 23.38 | 70.15 | 1516.95 | 2250.65 | < 0.001 | OSCC > NOM |
| M1/M | Stroma | 0.07 | 0.22 | 1.58 | 3.08 | < 0.001 | OSCC > NOM |
| M2/M | Stroma | 0.14 | 0.42 | 2.14 | 2.31 | < 0.001 | OSCC > NOM |
| M1/M2 | Stroma | 0.06 | 0.17 | 2.72 | 6.21 | < 0.001 | OSCC > NOM |
| CD4 | Epithelium | 1123.28 | 947.82 | 5388.66 | 2892.32 | < 0.001 | OSCC > NOM |
| CD8 | Epithelium | 58.94 | 14.03 | 51.50 | 14.40 | 0.191 | NOM > OSCC |
| FoxP3 | Epithelium | 40.76 | 85.97 | 122.01 | 114.58 | 0.012 | OSCC > NOM |
| CD20 | Epithelium | 490.34 | 380.15 | 461.68 | 510.15 | 0.578 | NOM > OSCC |
| CD66b | Epithelium | 40.79 | 12.47 | 38.80 | 11.80 | 0.378 | NOM > OSCC |
| PD-L1 | Epithelium | 7.86 | 19.26 | 1320.56 | 2332.16 | 0.100 | OSCC > NOM |

**Table S5** Correlations between expressions of various biomarkers in the stromal compartment of OSCC

| Marker | CD4 | | | CD8 | | | FoxP3 | | | CD20 | | | CD66b | | |
| --- | --- | --- | --- | --- | --- | --- | --- | --- | --- | --- | --- | --- | --- | --- | --- |
|  | χ² | Phi coefficient | *P* | χ² | Phi coefficient | *P* | χ² | Phi coefficient | *P* | χ² | Phi coefficient | *P* | χ² | Phi coefficient | *P* |
| CD4 |  | | | 24.70 | 0.83 | <0.001 | 0.66 | 0.14 | 0.418 | 15.80 | 0.66 | <0.001 | 18.29 | 0.71 | <0.001 |
| CD8 | 24.70 | 0.83 | <0.001 |  | | | 0.26 | 0.09 | 0.607 | 18.57 | 0.72 | <0.001 | 15.55 | 0.66 | <0.001 |
| FoxP3 | 0.66 | 0.14 | 0.418 | 0.26 | 0.09 | 0.607 |  | | | 0.82 | 0.15 | 0.364 | 1.44 | 0.20 | 0.230 |
| CD20 | 15.80 | 0.66 | <0.001 | 18.57 | 0.72 | <0.001 | 0.82 | 0.15 | 0.364 |  | | | 18.57 | 0.72 | <0.001 |
| CD66b | 18.29 | 0.71 | <0.001 | 15.55 | 0.66 | <0.001 | 1.44 | 0.20 | 0.230 | 18.57 | 0.72 | <0.001 |  | | |
| PD-L1 | 0.01 | 0.02 | 0.908 | 1.44 | 0.20 | 0.230 | 1.44 | 0.20 | 0.230 | 0.82 | 0.15 | 0.364 | 0.03 | -0.03 | 0.864 |
| M1/M | 0.02 | -0.03 | 0.878 | 0.21 | -0.08 | 0.650 | 0.05 | 0.04 | 0.821 | 0.36 | 0.10 | 0.549 | 0.05 | 0.04 | 0.821 |
| M2/M | 5.84 | -0.40 | 0.016 | 3.86 | -0.33 | 0.049 | 0.01 | 0.02 | 0.908 | 2.34 | -0.26 | 0.126 | 3.86 | -0.33 | 0.049 |
| M1:M2 | 1.19 | 0.18 | 0.275 | 2.26 | 0.25 | 0.133 | 0.01 | 0.02 | 0.908 | 1.50 | 0.20 | 0.221 | 4.82 | 0.37 | 0.028 |
| Marker | PD-L1 | | | M1/M | | | M2/M | | | M1:M2 | | |  |  |  |
|  | χ² | Phi coefficient | *P* | χ² | Phi coefficient | *P* | χ² | Phi coefficient | *P* | χ² | Phi coefficient | *P* |  |  |  |
| CD4 | 0.01 | 0.02 | 0.908 | 0.02 | -0.03 | 0.878 | 5.84 | -0.40 | 0.016 | 1.19 | 0.18 | 0.275 |  |  |  |
| CD8 | 1.44 | 0.20 | 0.230 | 0.21 | -0.08 | 0.650 | 3.86 | -0.33 | 0.049 | 2.26 | 0.25 | 0.133 |  |  |  |
| FoxP3 | 1.44 | 0.20 | 0.230 | 0.05 | 0.04 | 0.821 | 0.01 | 0.02 | 0.908 | 0.01 | 0.02 | 0.908 |  |  |  |
| CD20 | 0.82 | 0.15 | 0.364 | 0.36 | 0.10 | 0.549 | 2.34 | -0.26 | 0.126 | 1.50 | 0.20 | 0.221 |  |  |  |
| CD66b | 0.03 | -0.03 | 0.864 | 0.05 | 0.04 | 0.821 | 3.86 | -0.33 | 0.049 | 4.82 | 0.37 | 0.028 |  |  |  |
| PD-L1 |  | | | 0.82 | 0.15 | 0.364 | 2.26 | 0.25 | 0.133 | 0.01 | 0.02 | 0.908 |  |  |  |
| M1/M | 0.82 | 0.15 | 0.364 |  | | | 3.65 | 0.32 | 0.056 | 6.76 | 0.43 | 0.009 |  |  |  |
| M2/M | 2.26 | 0.25 | 0.133 | 3.65 | 0.32 | 0.056 |  | | | 1.03 | -0.17 | 0.311 |  |  |  |
| M1:M2 | 0.01 | 0.02 | 0.908 | 6.76 | 0.43 | 0.009 | 1.03 | -0.17 | 0.311 |  | | |  |  |  |

**Table S6** Correlations between expressions of various biomarkers in the epithelial compartment of OSCC

| Marker | CD4 | | | CD8 | | | FoxP3 | | | CD20 | | | CD66b | | | PD-L1 | | |
| --- | --- | --- | --- | --- | --- | --- | --- | --- | --- | --- | --- | --- | --- | --- | --- | --- | --- | --- |
|  | χ² | Phi coefficient | *P* | χ² | Phi coefficient | *P* | χ² | Phi coefficient | *P* | χ² | Phi coefficient | *P* | χ² | Phi coefficient | *P* | χ² | Phi coefficient | *P* |
| CD4 |  | | | 0.71 | 0.14 | 0.400 | 0.29 | 0.09 | 0.593 | 0.91 | -0.16 | 0.342 | 2.68 | -0.27 | 0.102 | 0.18 | -0.07 | 0.676 |
| CD8 | 0.71 | 0.14 | 0.400 |  | | | 0.56 | 0.13 | 0.453 | 5.36 | 0.39 | 0.021 | 2.70 | 0.27 | 0.101 | 0.14 | 0.06 | 0.709 |
| FoxP3 | 0.29 | 0.09 | 0.593 | 0.56 | 0.13 | 0.453 |  | | | 5.71 | 0.40 | 0.017 | 0.14 | 0.06 | 0.709 | 0.09 | 0.05 | 0.765 |
| CD20 | 0.91 | -0.16 | 0.342 | 5.36 | 0.39 | 0.021 | 5.71 | 0.40 | 0.017 |  | | | 0.42 | 0.11 | 0.516 | 4.10 | 0.34 | 0.043 |
| CD66b | 2.68 | -0.27 | 0.102 | 2.70 | 0.27 | 0.101 | 0.14 | 0.06 | 0.709 | 0.42 | 0.11 | 0.516 |  | | | 0.42 | -0.11 | 0.516 |
| PD-L1 | 0.18 | -0.07 | 0.676 | 0.14 | 0.06 | 0.709 | 0.09 | 0.05 | 0.765 | 4.10 | 0.34 | 0.043 | 0.42 | -0.11 | 0.516 |  | | |
